# Pilot-testing service-based planning for health care in rural Zambia

**DOI:** 10.1186/1472-6963-14-S1-S7

**Published:** 2014-05-12

**Authors:** Fastone M Goma, Gail Tomblin Murphy, Miriam Libetwa, Adrian MacKenzie, Selestine H Nzala, Clara Mbwili-Muleya, Janet Rigby, Amy Gough

**Affiliations:** 1 School of Medicine, University of Zambia, P.O.Box 50110, Lusaka, Zambia; 2 WHO/PAHO Collaborating Centre on Health Workforce Planning and Research, Dalhousie University, 5869 University Avenue, Halifax, Canada; 3 Ministry of Health, Republic of Zambia, Ndeke House, P. O. Box 30205, Lusaka, Zambia

**Keywords:** service-based planning, competencies, human resources for health, planning, policy, Zambia, planification fondée sur les services, compétences, ressources humaines en santé, planification, politiques, Zambie

## Abstract

**Background:**

Human resources for health (HRH) planning in Zambia, as in other countries, is often done by comparing current HRH numbers with established posts, without considering whether population health needs are being met. Service-based HRH planning compares the number and type of services required by populations, given their needs, with the capacity of existing HRH to perform those services. The objective of the study was to demonstrate the effectiveness of service-based HRH planning through its adaptation in two rural Zambian districts, Gwembe and Chibombo.

**Methods:**

The health conditions causing the greatest mortality and morbidity in each district were identified using administrative data and consultations with community health committees and health workers. The number and type of health care services required to address these conditions were estimated based on their population sizes, incidence and prevalence of each condition, and desired levels of service. The capacity of each district’s health workers to provide these services was estimated using a survey of health workers (n=44) that assessed the availability of their specific competencies.

**Results:**

The primary health conditions identified in the two districts were HIV/AIDS in Gwembe and malaria in Chibombo. Although the competencies of the existing health workforces in these two mostly aligned with these conditions, some substantial gaps were found between the services the workforce can provide and the services their populations need. The largest gaps identified in both districts were: performing laboratory testing and interpreting results, performing diagnostic imaging and interpreting results, taking and interpreting a patient’s medical history, performing a physical examination, identifying and diagnosing the illness in question, and assessing eligibility for antiretroviral treatment.

**Conclusions:**

Although active, productive, and competent, health workers in these districts are too few to meet the leading health care needs of their populations. Given the specific competencies most lacking, on-site training of existing health workers to develop these competencies may be the best approach to addressing the identified gaps. Continued use of the service-based approach in Zambia will enhance the country’s ability to align the training, management, and deployment of its health workforce to meet the needs of its people.

## Abstract (fr)

**Résumé:**

**Contexte**

En Zambie, comme dans d’autres pays, la planification des ressources humaines en santé consiste souvent à comparer les données sur les ressources et le nombre de postes établis, sans égard aux besoins de la population en matière de santé. La planification fondée sur les services consiste à comparer le nombre et le type de services requis par les populations, en fonction de leurs besoins, et la capacité des ressources existantes à fournir ces services. L’étude avait pour but de mettre en oeuvre la planification des ressources humaines en santé fondée sur les services dans deux districts ruraux de la Zambie – Gwembe et Chibombo – afin d’en démontrer l’efficacité.

**Méthodes**

Au moyen des données administratives et de la consultation des comités de santé communautaire et des travailleurs de la santé, on a cerné les problèmes de santé qui causent le plus de mortalité et de morbidité dans chaque district. Le nombre et le type de services de santé requis pour traiter ces problèmes ont été estimés en fonction de la taille de la population, de l’incidence et de la prévalence de chaque problème et des niveaux de service souhaités. La capacité des travailleurs de la santé de chaque district à fournir ces services a été estimée grâce à une enquête menée auprès de ces travailleurs (n = 44), qui a évalué leurs compétences particulières.

**Résultats**

Les principaux problèmes de santé relevés étaient le VIH/sida dans le district de Gwembe et le paludisme dans le district de Chibombo. Si les compétences des travailleurs de la santé de ces deux districts cadraient en grande partie avec ces problèmes, d’importants écarts ont été décelés entre les services fournis par ces travailleurs et les services dont ont besoin les populations qu’ils servent. Les écarts les plus importants qui ont été relevés dans les deux districts avaient trait aux éléments suivants : les tests en laboratoire et l’interprétation des résultats, l’imagerie diagnostique et l’interprétation des résultats, la vérification et l’interprétation des antécédents médicaux des patients, l’examen physique, le dépistage et le diagnostic de la maladie et l’évaluation de l’admissibilité au traitement antirétroviral.

Bien qu’ils soient actifs, productifs et compétents, les travailleurs de la santé de ces districts sont trop peu nombreux pour répondre aux principaux besoins de la population en matière de santé. Compte tenu des compétences particulières où les lacunes sont les plus grandes, offrir une formation en milieu de travail aux travailleurs de la santé actuels afin qu’ils acquièrent ces compétences pourrait être la meilleure approche pour réduire les écarts qui ont été relevés. L’utilisation continue de cette approche fondée sur les services en Zambie améliorera la capacité du pays à penser la formation, la gestion et le déploiement de ses travailleurs en santé en fonction des besoins de sa population.

## Background

Human resources for health (HRH) constitute a key component of any national health system, and Zambia is currently experiencing an HRH crisis [1] due to the shortage of trained health workers, particularly in rural and remote areas. As of 2010, there were 27 728 health workers in Zambia to fill 51 404 posts, representing a gap of nearly 50% [2,3]. A recent study suggests that even with no further losses of existing health workers (HWs) and full employment of new graduates, staffing targets for doctors, nurses, and clinical officers will not be met by 2018 [4]. The 2008 shortfalls for selected HWs countrywide stood at 46% for medical doctors, 53% for midwives, 60% for clinical officers, 46% for nurses, 64% for pharmacists, and 47% for environmental health staff [2]. Each of these shortages necessitates multi-tasking and task-shifting by existing health workers, among other coping strategies [2].

Despite Zambia’s efforts to resolve the HRH crisis [5] and address key issues such as the production of more skilled HWs to deliver health services, the training of HWs has not kept pace with health sector needs, especially to address the increasing burden of disease as a result of HIV/AIDS [2] and to cater to the evolving and expanding HW roles and new forms of service provision. One of the key strategies toward addressing this crisis is to plan for the health workforce in a way that aligns the services that health care providers are able to perform with the specific health care needs of the populations being served. However, in Zambia as in many other countries, HRH planning is often done by comparison of the number of HRH with established posts or HRH to population ratios [6]. Zambia’s Ministry of Health (MoH) has noted, however, that the current number and distribution of these posts may not be adequately aligned with population health needs [2]. The quantities and distributions of established posts approved by Zambia’s Cabinet Office are solely based on population-to-provider ratios [2] which do not allow for the consideration of differences in health needs or care delivery modalities across populations or over time [6-8].

To respond to the needs of people, policy and decision makers must consider the number and type of services that will be required to address those needs and the competencies (i.e., the knowledge, skills, and judgements) required of health care providers to deliver those services. A service-based approach^i^[9,10] to HRH planning that directly incorporates each of these factors in estimating HRH requirements and supply, and subsequently identifies gaps between the two, allows policy makers to plan health care more effectively for the population. Such an approach allows policy makers to a) identify, and b) determine strategies or policies to address health care system gaps at the level of specific services rather than particular professions. In this way it is designed to offer greater flexibility than more conventional, profession-specific planning.

The objective of this research was to demonstrate the effectiveness of a service-based approach to HRH planning through its adaptation in two rural Zambian districts, Gwembe and Chibombo.

Chibombo is located in Zambia’s Central Province and has a population of approximately 294 000 [9] spread over 13 670 km^2^ – just under 22 people per square kilometre. The district’s leading cause of mortality is HIV/AIDS, which accounts for 20% of all deaths. When the study was conducted there were 136 trained health workers in the district (a ratio of over 2 100 persons per health worker) to staff 24 rural health centres, one remote health post, and one district hospital. There are no private health care facilities in Chibombo.

Gwembe is a district in Southern Province, with a population of approximately 53 000 [9] spread over 12 600 km^2^ – fewer than five people per square kilometre. Malaria incidence is approximately 52%. At the time of the study there were 50 trained health workers in the district (over 1 000 persons per health worker) to staff eight rural health centres, one district hospital, and a ninth health centre affiliated with the hospital. Gwembe has no private health care facilities.

This work was part of a larger research study [12] that also evaluated existing HRH retention and recruitment strategies for health workers in the two districts named above.

## Methods

**Figure 1:**
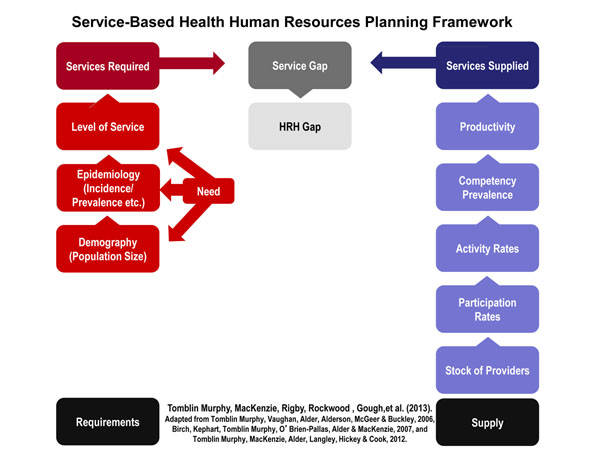
Service-based health human resources planning framework

This research was carried out using a framework for service-based planning [9,10] depicted below in Figure 1. The unit of analysis used in this framework is the particular health care service that must be performed by a health care provider to address a certain health care need within the target population. This contrasts with most HRH planning approaches where the health care provider is the unit of analysis. The framework, depicted in Figure 1, calls for two quantities to be estimated: how often a particular service is required by the target population (requirements), and how often that service can be performed by the available health workforce (supply). Estimating the first requires the identification of the leading health conditions which drive the need for health care (according to whatever criteria are deemed appropriate by planners in their particular context, such as rates of mortality and/or morbidity), knowledge of the size of the population, and the incidence or prevalence of each leading condition within it, the range of health care services required to address each condition, and the frequency with which each service is required by persons with those conditions. In addition to aligning services with the health needs of the population, consultation and engagement with key stakeholders allows for the consideration of contextual factors impacting this alignment, such as barriers and enablers to accessing services. In this way, planners can explicitly consider issues of access or inequalities in how services are organized and provided.

In addition to reviewing the leading causes of mortality according to administrative records in each district, a set of stakeholder focus groups was conducted in each district. There was one focus group with health workers, another with the community health boards, and a third with representatives from both groups (for a total of six focus group discussions across the two districts). The focus groups that included community members used local languages (Nyanja in Chibombo and Tonga in Gwembe) for clarity as needed. The purpose of these focus groups, which used a modified Delphi approach, was to obtain consensus from the participating stakeholders as to which health condition they felt was most burdensome in terms of mortality and morbidity experienced by the people in the districts. In this pilot application, two leading health conditions (one for each district) were identified – HIV/AIDS in Chibombo and malaria in Gwembe. These were the conditions perceived by participants as being the most burdensome – in terms of morbidity as well as mortality – for their communities.

Next, lists of the competencies required by the health workforce to manage patients with each condition were compiled. This process began with a draft list based on the standards of care for each condition from the Integrated Technical Guidelines published by the World Health Organization’s Regional Office for Africa [13]. This initial list was amended by the project team, and then finally validated by Zambian clinicians with expertise in management of those conditions in the Zambian context.

The epidemiological component of the framework – i.e. the incidence of malaria and the combined incidence and prevalence of HIV/AIDS among district residents – was obtained from district-level administrative data. The expected distribution of patients by care setting (health centre, district hospital), as well as the proportion of ill patients requiring each service within those settings, were estimated through consultations with administrators and clinicians in each district, supplemented by administrative records on the volumes of service use in each setting. The latter source was deemed insufficient on its own to estimate appropriate service levels because the known shortage of HRH meant that existing service levels underestimate those that are actually needed. These two points of information (distribution of patients by care setting and proportion in each care setting requiring the competency) reflect the level of service component of the framework – that is, the number and type of health care services patients in these districts should receive given that they have either of these conditions (i.e. are deemed to medically require and would receive services if sufficient resources were available).

To obtain the data needed to estimate the supply of services in the two districts, a questionnaire for HWs was developed asking about the following:

• Whether they provided direct patient care (participation level or proportion)

• Whether they worked part-time or full-time (activity level)

• Whether they felt they had the knowledge, skills, and judgement to safely perform each of the services listed without supervision (perceived competency prevalence)

The questionnaire was adapted from previous work by the research team [9,10], and was pilot-tested with a select group of professionals in a different district, Chongwe. Ethical approval for the research study was obtained from the University of Zambia and Dalhousie University research ethics boards. Questionnaires were completed for each respondent by one of four interviewers, working simultaneously, in each district. Interviews were conducted in English. Participants confirmed their responses as they were recorded to ensure accuracy.

To obtain the perspectives of health workers practicing in different conditions, in each district, health facilities selected for the interviews included one rural health centre, one remote health centre, and the district hospital. Limitations on time and resources for the study prohibited travelling to every health centre in both districts. The questionnaire was administered to all HWs that could be located. A total of 44 HWs completed the questionnaire. Administrative data from the district offices indicated that, across both districts, there were just under 50 HWs practicing at these facilities, out of 186 in total across the two districts. There is some uncertainty as to actual facility-specific numbers because some HWs who are nominally posted to one facility may in fact practice at another where they are deemed to be more urgently needed.

As shown in Table 1, most respondents (67%) were aged between 20 and 40 years, and most (75%) held a professional diploma or certification as their highest level of formal education. Males and females, and workers from the two districts, had roughly equal representation at close to 50% each. Nurses were the most common profession in the sample, accounting for 40% of respondents, followed by midwives at 14%.

**Table 1:** Sample characteristics

Attribute	Category	Percentage	Number
Age	<20 30-39 40-49 50-59 60+	0% 38% 29% 18% 13%	0 18 13 8 5

Gender	Female Male	51% 49%	23 21

Profession	Clinical Officer Doctor Environmental Health Technologist (EHT) Midwife Nurse Pharmacist Other	11% <5% <5% 14% 40% <5% 13%	5 - - 6 18 - 6

Education	Secondary school only Professional certificate/diploma Bachelor’s degree Missing	11% 75% <10% <5%	5 33 <5 <5

District	Chibombo Gwembe	49% 51%	21 23

Cell counts less than five have been suppressed to protect the anonymity of participants. “Other” included laboratory technicians and assistants, pharmacy technicians, nutritionists, physiotherapists, and radiographers, all of whom would have at least some face-to-face interactions with patients.

The supply of and requirements for each competency were compared and gaps in specific competencies were identified for Chibombo and Gwembe. To use the example the amount of times in a typical week screening for eligibility for antiretroviral therapy (ART) would be required in Chibombo, the size of the district population is multiplied by the combined incidence and prevalence rate for HIV/AIDs to yield the number of people who might require treatment. Multiplying this value by the estimated proportion who would require ART screening per week yields the estimated number of times that service is required in a typical week. To estimate how often Chibombo’s existing health workforce could potentially provide ART screening in a typical week, the number of health workers in the district is multiplied by the estimated proportion who are active in direct patient care (participation level), the proportion of full-time hours they work (activity level), the estimated proportion who feel competent to perform that service (perceived competency prevalence), and the estimated number of HIV/AIDS patients the district as a whole can treat per week (productivity).

Similarly, to estimate the amount of times in a typical week intravenous medications would be required in Gwembe, the size of the district population is multiplied by the malaria incidence rate to yield the number of people who might require that treatment for malaria. Multiplying this value by the estimated proportion of malaria patients who would require IV treatment per week yields the estimated number of times that service is required in a typical week. To estimate how often Gwembe’s existing health workforce could potentially provide IV treatment in a typical week, the number of health workers in the district is multiplied by their estimated levels of participation and activity, the prevalence of the perceived prevalence to provide that service, and their productivity.

In this way, the number of times each service may be required by the population in each district was estimated and compared with estimates of the number of times it could be provided by the health workforce in each district, and gaps – i.e. differences in these two figures – estimated for each service. In parallel with these activities, there was a process of expert and key informant consultation in both Chibombo and Gwembe to link the list of competencies with the health workers that can perform them according to legal and regulatory frameworks in Zambia. With the combination of identified competency gaps and linkages between these competencies and the workers that can perform them, research and policy-making partners worked together to draft potential policy strategies tailored to address shortages of specific competencies by drawing on the full range of health care professions and professionals with those specific competencies in each jurisdiction.

## Results

Administrative data from the district offices suggested that cardiovascular and respiratory illness, respectively, were the leading causes of hospitalization in the districts. Other leading conditions were HIV/AIDS, malaria, diseases of the eye, and diarrheal diseases. These data were similar to health workers’ experiences and community perspectives, described in the focus groups, as far as which conditions were the most pressing, however their relative rankings differed. From the perspectives of the clinicians and community representatives, while the other conditions were identified as problems, HIV/AIDS (in Chibombo) and malaria (in Gwembe) were the conditions that placed the greatest burden on their communities and their health care systems.

In discussing this apparent discrepancy, representatives from the Ministry of Health as well as the districts identified several possible contributing factors. The cases that are referred to hospitals are not always the most severe – rather they are the ones in which hospital care is expected to be of most benefit; for example, a severely but terminally ill patient may not be sent to hospital. Similarly, conditions that only rarely become severe, such as malaria, may nonetheless result in a considerable burden on patients and the health care system even though hospitalization does not occur. In addition, most deaths in the districts occur outside the hospital, and in such cases causes of death are often not able to be formally recorded because physicians are rarely present; hence administrative data on mortality due to specific conditions are likely underestimates.

After considering this information, a combination of the administrative and community data identified the leading health conditions in Chibombo and Gwembe as HIV/AIDS and malaria, respectively. These choices were subsequently validated by each district’s chief medical officer and other health workers.

Data provided through the HW questionnaires were analyzed using Microsoft Excel to estimate the supply of each service in each district. All respondents reported participating in direct patient care, and working full-time hours (i.e., in terms of the analytical framework, their participation and activity levels were 100%). Indeed, in focus group discussions it was reported that HWs are often compelled to work longer than full-time hours in response to the needs of their patients. In addition, district managers reported that their respective workforces were able to treat nearly 27 000 malaria cases (in Gwembe) and over 19 000 patients with HIV/AIDS (in Chibombo) in the past year.

For each service, the estimated number of times it could be provided per week by the existing district workforce (supply) was compared to the estimated number of times it would be required per week by the district population (requirements), and gaps identified. The services for which the gaps were largest – i.e. those for which the difference between the estimated number of times the service is required and the number of times it can be provided by the existing workforce were greatest – were identified in both districts. These gaps were validated by the participating health workers as being consistent with their experiences, and are summarized in Tables 2 and 3. Although the districts and their leading health conditions are different, there were several similarities in the services they found to be in short supply.

**Table 2:** Largest estimated services gaps in Chibombo

Service	Estimated number of times service is required/week	Estimated number of times service can be provided/week	Estimated gap
Interpret the results of history, physical exam, chest x-ray, and lab tests, leading to a diagnosis	4 732	81	-4 651

Screen for eligibility for antiretroviral treatment/prophylaxis	4 732	111	-4 621

Perform a physical examination	4 732	132	-4 600

Obtain consent for antiretroviral treatment/prophylaxis	4 732	132	-4 600

Take a screening history of the chief complaint and relevant aspects of past medical history, current medications, etc.	4 732	152	-4 580

**Table 3:** Largest estimated services gaps in Gwembe

Service	Estimated number of times service is required/week	Estimated number of times service can be provided/week	Estimated gap
Perform clinical laboratory testing service (haematology, chemistry, RDT, MP slides, etc.)	27 197	17 965	-9 232

Triage patients according to acuity of illness and need for care, and refer to appropriate care setting (primary and secondary assessment, hospital, emergency department, non-traditional care site, community)	27 197	22 457	-4 740

Order clinical diagnostic tests	27 197	25 451	-1 746

Perform a physical examination	27 197	26 948	-249

Interpret the results of history, physical exam, and lab tests, etc., leading to a diagnosis	27 197	26 948	-249

The magnitude of the service gaps was larger in Chibombo, mainly due to its larger population. Two of its five largest service gaps were for services that would be required only by patients with HIV/AIDS: screening for antiretroviral treatment eligibility, and obtaining consent for same. In Gwembe, however, none of the largest service gaps were for services uniquely required by persons suffering from malaria – each of those services would also be used to treat a range of other conditions. In both districts, the gaps for these services were largest because they are required very often yet few HWs in either district report feeling competent to perform them – a combination of high need and low supply.

## Discussion

Of the largest service gaps identified in Chibombo and Gwembe, some are the domain of only a few professional cadres, which are in especially short supply in Zambia. Performing physical exams, and interpreting exam and test results leading to a diagnosis, for example, can only be performed by a doctor or clinical officer, according to Zambia’s regulatory guidelines [16,17]. Addressing the shortage of such services is thus likely to be difficult, requiring targeted recruitment of such professionals (already a great challenge [1-3]) or perhaps advanced training of other professionals and the associated regulatory accommodation; neither option is easily achieved.

However, many of these services are within the regulatory domains of multiple cadres that are not as scarce as doctors. Taking screening histories, triaging patients, ordering lab tests, and obtaining informed consent for treatment are all services within the domains of registered and enrolled nurses as well as midwives. Some targeted professional development training to refresh these competencies among existing members of these cadres might therefore go a long way toward alleviating these gaps in the short term. Focus group discussions with members of these professions in these districts indicate that such training would be welcomed. In the longer term, perhaps more emphasis on these specific competencies in pre-licensure training would help to reduce their scarcity among a range of health professions. The further step of conducting this sort of competency/service gap analysis on a regular basis would help to inform ongoing refinements to the various curricula of different health professions.

The study was limited by the small number of HWs available to participate, and the use of self-reported data for important measures such as competency. Although more objective measures of competency exist, the study lacked the time and resources to apply these, necessitating the reliance on participants’ self-assessments. Further, the study is specific to the two leading conditions in the districts, although of course there are others that require treatment as well – this underscores the service gaps that have been identified. Although these features may have limited the accuracy of the findings, they were in fact validated by the participating HWs as well as representatives of the Ministry of Health and other Zambian stakeholders through several engagement activities. One such type of engagement included a deliberative forum where the findings were shared with a group of key stakeholders who then validated the findings and contributed to the development and refinement of subsequent recommendations to the MoH and other relevant government entities (e.g., Ministry of Community Development, Mother and Child Health; Ministry of Finance; Ministry of Education; Cabinet Office; World Health Organization Country Office; Clinton Health Access Initiative).

The participants in this forum discussed the numerous challenges to recruitment and retention of the types of health professionals who normally possess the missing competencies. As such, addressing the identified gaps by increasing the supply of HWs who have acquired them in their pre-service training would likely prove difficult – attracting and retaining doctors and nurses to practice in rural areas is something Zambia’s government has devoted considerable effort and resources to, with very limited success [1,2]. It was therefore agreed that addressing these gaps would likely take the form of increasing the level of competence in the HWs already practicing in these districts. Hence one of the resulting recommendations from the deliberative forum was that targeted in-service training delivered in Gwembe and Chibombo may be the most effective short-term solution to address the identified gaps. In the longer term, continual use of needs- and service-based assessments should be used to inform Zambia’s health professional education and training institutions as they continuously adapt their pre-service curricula according to desired graduate competencies. The continued use of needs- and service-based assessments should also be used to inform on-going HRH planning, evaluation, and monitoring, including task-shifting guidelines, HRH hiring and recruitment, induction and orientation, and continuing education.

Although this pilot application has focused on two leading health conditions - largely because of limitations to time and resources - the intent of the service-based planning method is not to promote a disease-centric approach to HRH or health system planning. Rather it is hoped that this study will serve to demonstrate the utility of the approach in principle, thereby promoting its application across the range of health conditions affecting the populations being served. Indeed, as noted above, the largest gaps found in this study tended to be in services that are required to treat many different health conditions.

## Conclusions

Although active, productive, and competent, health workers in these districts are too few to meet the leading health care needs of their populations. Given the specific competencies most lacking, on-site training of existing health workers may be the best short-term approach to addressing this gap. In the long term, continued use of the service-based approach in Zambia will enhance the country’s ability to align the training, management, and deployment of its health workforce to meet the needs of its people. This approach will provide the necessary flexibility to change the composition of teams to align with the changing health needs of the people. The application of the service-based framework in this setting and the relevance of findings to dialogue with policy makers and clinical stakeholders demonstrate its potential utility for other jurisdictions and health care settings internationally.

## List of abbreviations

DFATD: Foreign Affairs, Trade and Development Canada; GHRI: Global Health Research Initiative; HRH: Human resources for health; HW: Health worker; IDRC: International Development Research Centre; MoH: Ministry of Health

## Competing interests

The authors have no competing interests to declare.

## Authors' contributions

FMG, GTM, AM, and SN led the conceptualization, data collection, interpretation of findings, writing, and editing. ML led the engagement and communication with key stakeholders and decision-makers from MoH throughout the project and contributed to the conceptualization, data collection, interpretation of findings, writing, and editing. CMM contributed to the conceptualization, interpretation of findings, writing, and editing. AG contributed to data collection, interpretation of findings, writing, and editing. JR contributed to writing and editing. All authors have read and approved the final manuscript.

## Endnotes

i. This approach was previously referred to as a “competency-based approach” (see Tomblin Murphy et al., 2012) because of the measurement of health care provider competencies required to deliver services that meet the health care needs of people; however, the term has been changed to “service-based” to better reflect the emphasis of the approach on the health needs of people rather than planning based on the needs of providers.

## Declaration

The publication costs associated with this article are funded by Foreign Affairs, Trade and Development Canada and the International Development Research Centre through the Global Health Research Initiative.

This article has been published as part of *BMC Health Services Research* Volume 14 Supplement 1, 2014: Uptake and impact of research for evidence-based practice: lessons from the Africa Health Systems Initiative's research component. The full contents of the supplement are available online at http://www.biomedcentral.com/bmchealthservres/supplements/14/S1
